# Protocol for genome-wide CRISPR knockout screens of bacterial cytotoxins in HeLa cells

**DOI:** 10.1016/j.xpro.2022.101595

**Published:** 2022-07-31

**Authors:** Qi Yang, Yao Zhou, Liuqing He, Yuanyuan Zhang, Liang Tao

**Affiliations:** 1 College of Life Sciences, Zhejiang University, Hangzhou, Zhejiang 310058, China; 2 Key Laboratory of Structural Biology of Zhejiang Province, School of Life Sciences, Westlake University, Hangzhou, Zhejiang 310024, China; 3 Center for Infectious Disease Research, Westlake Laboratory of Life Sciences and Biomedicine, Hangzhou, Zhejiang 310024, China

**Keywords:** Sequence analysis, Cell Biology, Cell culture, Sequencing, High Throughput Screening, Microbiology, Molecular Biology, CRISPR

## Abstract

CRISPR screening is a powerful tool to identify host factors for pathogenic agents including viruses and bacterial toxins. Here, we present a protocol to conduct a genome-scale CRISPR screen on HeLa cells for host factors involved in the toxin action of *Clostridioides difficile* TcdB4. We describe in detail how to prepare the library, set up the screen, obtain the gene sequences, and analyze the results. This protocol can also be modified for other genome-scale libraries, cell lines, and cytotoxins.

For complete details on the use and execution of this protocol, please refer to Luo et al. (2022).

## Highlights

• A compatible protocol to perform genome-wide CRISPR knockout screens in HeLa cells
• Detailed steps for generating CRISPR knockout library in HeLa cells
• Estimation of appropriate toxin concentration for multi-round screening
• Sequencing using Illumine platform and CRISPR screen data processing

## Abstract (editor highlights)

Publisher’s note: Undertaking any experimental protocol requires adherence to local institutional guidelines for laboratory safety and ethics.

## Graphical abstract

**Figure:**
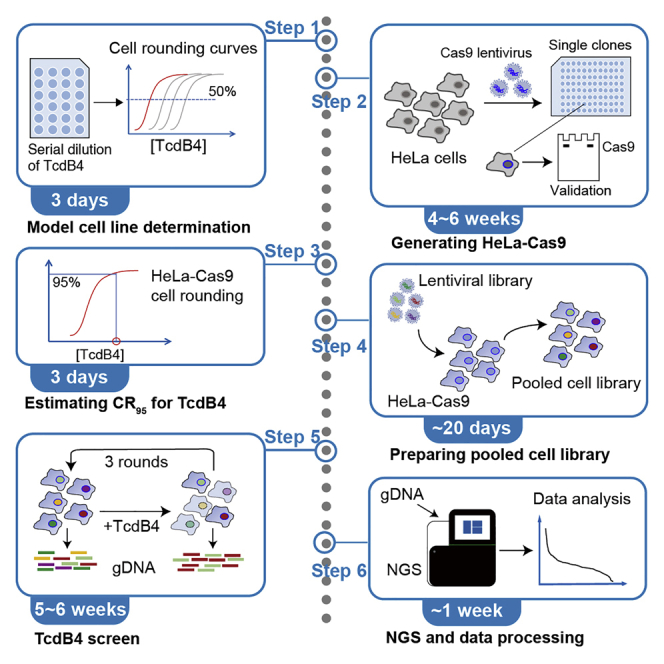

## Before you begin

The protocol below describes the CRISPR knockout screen for TcdB4 on HeLa cells using the GeCKO v2 library. However, we have also used this protocol in other human cell lines and with other genome-wide CRISPR libraries. To minimize possible biases, we strongly suggest using one batch of the toxin throughout the screen. Bacterial toxins can be divided into small aliquots and stored at −80°C for one year without overt loss of activity. The toxin should be used immediately after being thawed, and never refreeze and/or reused thawed toxin aliquots. Earlier versions of this protocol were applied for targeted screens with other bacterial cytotoxins (Tao et al., 2016, 2019; Zhou et al., 2021). For complete details on the use and execution of the protocol, please refer to (Luo et al., 2022).

### Model cell line determination

**Timing: 3 days**

Below we provided a detailed step-by-step protocol for testing the sensitivity of multiple cell lines to the target toxin (here TcdB4). The determined cell line is adopted for the CRISPR screen for TcdB4.

1. Grow candidate cell lines. After the trypsin digestion, collect and dilute the cells to appropriate concentrations with the respective medium. Seed each cell line to a 24-well plate and allow them to grow to a ∼60% confluent before adding the toxin. The inoculating density for each cell line may be different.

***Note:*** The choice of the model cell line is critical to performing the CRISPR screen, as resistant cells possibly lack the host factors for the toxin actions. In this protocol, we tested five cell lines including HeLa, U2OS, A549, MCF-7, and HT-29.

2. Prepare serial dilutions of the toxin (here TcdB4) within DMEM complete (DMEM supplemented with 10% FBS, 50 units/mL of penicillin, and 50 μg/mL of streptomycin). Dilute the stocked TcdB4 to 1 μM as a starting concentration.
3. Perform the serial dilution with a dilution factor of three. Prepare a total of 23 dilutions and final concentrations of TcdB are the following: 1 μM, 0.33 μM, 0.11 μM, 0.037 μM, 0.012 μM, 4 nM, 1.4 nM, 0.46 nM, 0.15 nM, 0.051 nM, 0.017 nM, 5.6 pM, 1.9 pM, 0.63 pM, 0.21 pM, 0.070 pM, 0.023 pM, 0.0077 pM, 0.0026 pM, 0.00086 pM, 0.00029 pM, and 0.0001 pM.
4. Remove the spent medium of the cell culture in the 24-well plate and add 0.5 mL of toxin dilution to each well. Leave one well of cells with the fresh medium as an untreated control. Put the plates back into the cell culture incubator.
5. After 14 h, capture the bright-field images of each well under the microscope. Manually count the numbers of round-shaped and normal-shaped cells (Figure 1A), calculate the cell rounding ratio, and plot them onto the toxin dose-cell rounding curves (Figure 1B).

**Figure 1:**
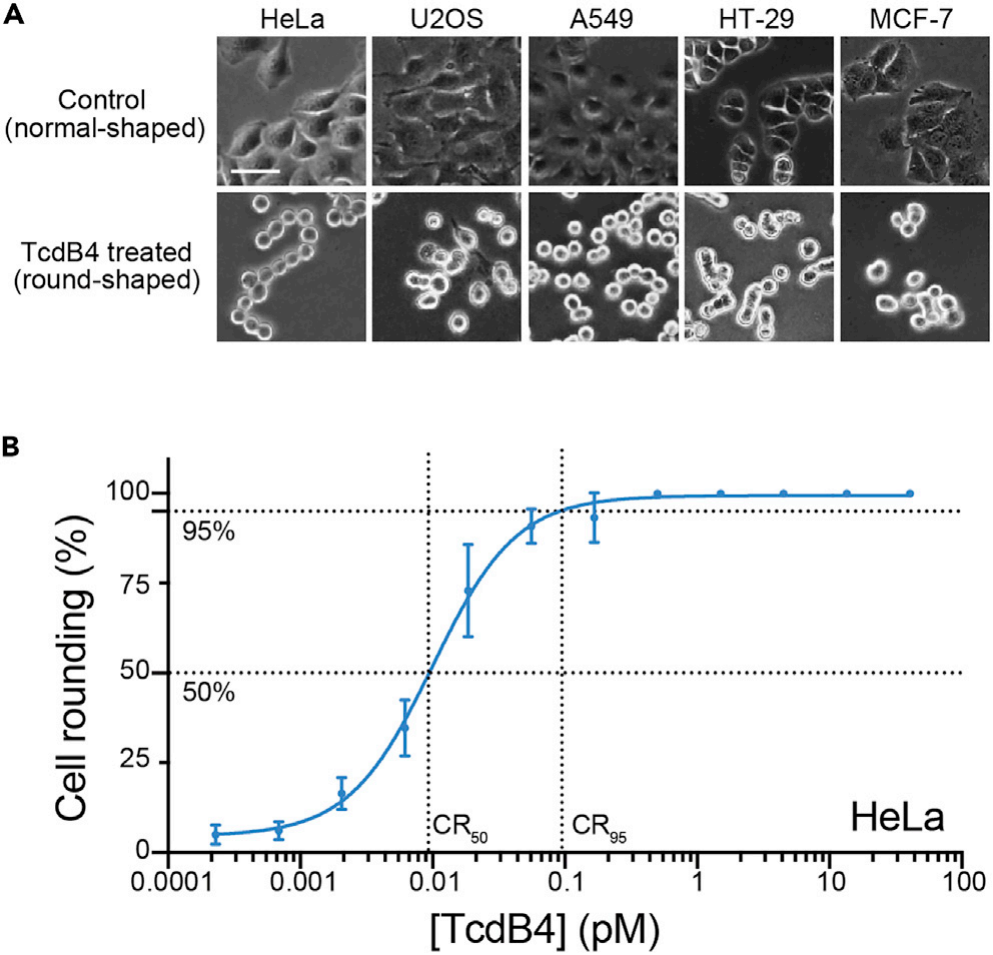
TcdB4-induced cytopathic effects as the readout for the screen (A) Microscopy images show normal HeLa, U2OS, A549, HT-29, and MCF-7 cells and cells treated by TcdB4 (round-shaped). The scale bar represents 50 μm. (B) The measured TcdB4 concentration-cell rounding chart for HeLa cells. Data are presented as mean ± standard deviation (SD) (n = 6).
6. Calculate the CR_50_ (toxin concentration that induces 50% of cell rounding) from the cell rounding curves for each cell line (Figure 1B). Cell lines with low CR_50_ value would be ideal model cells for the CRISPR screen. Here, HeLa cells are sensitive to TcdB4 and thus were chosen for the screen.

### Generating HeLa-Cas9 cell line

**Timing: 4–6 weeks**

We utilize lentivirus to integrate the *Streptococcus pyogenes cas9* gene together with the blasticidin resistance gene into the genome of HeLa cells.

**Figure 2:**
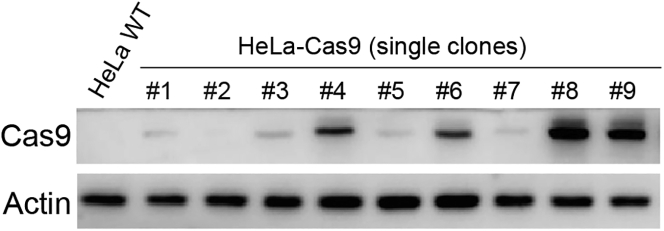
Validation of Cas9 expression The Cas9 expression levels in different single clones of HeLa-Cas9 cells were detected by immunoblot analysis. Actin is a loading control.

7. Preparing Cas9 lentivirus.
  a. Seed 2 × 10^6^ HEK293T cells into a 10 cm dish with the antibiotic-free DMEM complete. Grow the cells at 37°C with 5% CO_2_. The cells should achieve 80% confluency after 24 h of incubation.
  b. The transfection using PolyJet transfection reagent is performed following the manufacturer’s protocol (https://signagen.com/DataSheet/SL100688.pdf). The amounts of transfection reagent and plasmids are used as follows: set up a mixture of plasmids: 0.25 mL DMEM (serum-free), 3 μg psPAX2, 0.3 μg pMD2.G, and 3 μg pLenti-cas9-blasticidin; set up a transfection agent mixture: 0.25 mL DMEM (serum-free) and 18 μL of Polyjet.
  c. At 18 h post-transfection, replace the medium with 20 mL of fresh DMEM complete. Grow the cells for additional 30 h at 37°C in 5% CO_2_.
    ***Note:*** At this step, all operations should be as gentle as possible, as 293T cells may easily be detached from the plate.
  d. At 48 h post-transfection, collect the culture medium containing lentiviruses to a 15 mL sterile centrifuge tube (low retention).
    ***Optional:*** To completely remove the detached cells and cell debris, centrifuge the tube at 500 g for 5 min. Then filter the supernatant through a 0.45 μm syringe filter to a new 15 mL centrifuge tube.
  e. Split the lentiviral solution into 1 mL aliquots in the 1.5 mL microcentrifuge tubes. Store the lentivirus in the −80°C freezer.
    **CRITICAL:** When working with lentivirus, specific safety procedures must be followed. All materials including cell cultures, cell culture materials, and virus stocks that have contacted lentivirus must be bleached for 15 min before disposal.

8. Lentivirus infection.
  a. Seed the HeLa cells at a density of 5 × 10^5^ cells/mL onto a 6-well plate and incubate at 37°C in 5% CO_2_. The cells should achieve ∼80% confluency after 24 h of incubation.
  b. Dilute the lentiviral stock to the DMEM complete with dilution factors of 2, 6, 18, 54, and 162. Add the diluted lentivirus into the cell culture dropwise and gently rock the plate three times. Leave one well of the cells without lentivirus infection as an uninfected control. Place the culture plate back in the incubator.
  c. At 24 h post-infection, replace the culture medium with fresh DMEM complete containing 5 μg/mL Blasticidin.
  d. At 72 h post-infection, remove the culture medium and wash the cells with PBS three times. Add 2 mL of fresh DMEM complete with 2.5 μg/mL Blasticidin and continue the cultivation for additional 48 h. At this point, you should find that all cells in the uninfected control well are dead. For wells with infected cells normally grow under Blasticidin selection, pick the one with the highest viral dilution factor for following single colony isolation.
9. Single colony isolation and validation.
  a. Use trypsin to digest the cells, count the cell number, and dilute to a density of 3 cells/mL in the DMEM complete. Transfer 200 μL of diluted cells into a 96-well plate. Put the plate back in the cell culture incubator.
  b. Check 96-well plate under a microscope after one week. Mark the wells with only one colony formed, and replace the DMEM complete media.
  c. Allow the cells to grow for another week and then passage colonies to the 6-well plates. Pick up at least 10 colonies.
  d. Once the cells reach ∼80% confluency, freeze stock the cells of each colony as backups, and passage the cells to new 6-well plates for validation.
  e. Use RIPA to lyse the cells for each colony, load the lysate onto an SDS-PAGE, transfer it to a nitrocellulose membrane, and blot it with an anti-Cas9 antibody. The uninfected HeLa cells serve as the negative control (Figure 2). Save the colonies with robust Cas9 expression and select one for the following study.

### Estimating CR_95_ for TcdB4 on HeLa-Cas9

**Timing: 3 days**

Below we provided a detailed step-by-step protocol for estimating the CR_95_ for TcdB4 on HeLa cells. The CR_95_ value would be a reference toxin concentration when setting up the formal CRISPR screen.

10. Seed the HeLa-Cas9 cells with a density of 7 × 10^4^ to a 24-well plate and incubate at 37°C with 5% CO_2_ for 24 h. The cells should reach ∼60% confluency.
11. Thaw one vial of TcdB4 stock, dilute the toxin with DMEM complete to 30 pM as a starting concentration, and perform the serial dilution with a dilution factor of 3. Twelve dilutions are prepared.
12. Remove the spent medium and add 0.5 mL of toxin dilution to each well. Put the plates back into the cell culture incubator.
13. After 14 h, capture bright-field images for each well under the microscope. Count the numbers of round-shaped and normal-shaped cells, calculate the cell rounding ratio, plot them onto a toxin concentration-cell rounding chart, and align the cell rounding curve.
14. Calculate the CR_95_ from the cell rounding curve for HeLa-Cas9 (Figure 1B). This CR_95_ value would be a reference concentration on the screen.

## Key resources table

**Table:** 

REAGENT or RESOURCE	SOURCE	IDENTIFIER
**Antibodies**		

SpCas9 Antibody Clone 14B6 (1:3000)	GenScript	Cat# A01936

**Chemicals, peptides, and recombinant proteins**		

Puromycin	BBI	Cat# A610593-0025
Blasticidin S	Thermo Fisher Scientific	Cat# R21001
TcdB4	(Pan et al., 2021)	N/A
PolyJet	SignaGen	Cat# SL100688
2 × Hieff PCR Master Mix	Yeasen	Cat# 10102ES03

**Critical commercial assays**		

Blood & Cell Culture DNA Mini Kit	QIAGEN	Cat# 133223
GoldHi EndoFree Plasmid Maxi Kit	CWBIO	Cat# CW2104XQ
DNA Gel Extraction Kit	Axygen	Cat# AP-GX-250
NEB Next® UltraTM DNA Library Prep Kit for Illumina	NEB	Cat# E7370

**Experimental models: Cell lines**		

Human: HeLa	ATCC	CRL-1958; RRID: CVCL_3334
Human: 293T	ATCC	CRL-3216; RRID: CVCL_0063
Human: U2OS	ATCC	HTB-96; RRID: CVCL_0042
Human: A549	ATCC	CRM-CCL-185; CVCL_0023
Human: MCF-7	ATCC	HTB-22; CVCL_0031
Human: HT-29	ATCC	HTB-38; CVCL_0320

**Oligonucleotides**		

lentiGP-1_F: AATGGACTATCATATGCTTACCGTAACTTGAAAGTATTTCG	(Luo et al., 2022)	N/A
lentiGP-1_R: TAAAAAAGCACCGACTCGGTGCCACTTTTTCAAG	(Luo et al., 2022)	N/A

**Recombinant DNA**		

psPAX2	Addgene	Cat# 12260
pMD2.G	Addgene	Cat# 12259
GeCKO v2 (gRNA pooled library in lentiGuide-Puro + lentiCas9-Blast plasmid)	Addgene	Cat# 1000000049

**Software and algorithms**		

Cutadapt version 4.0	(Martin, 2011)	https://github.com/marcelm/cutadapt.git

## Step-by-step method details

### Generating human GeCKO v2 lentiviral library

**Timing: 6 days**

Below we provided a detailed step-by-step protocol for generating the human GeCKO v2 lentiviral library from the purchased human GeCKO v2 plasmid library.

1. Human GeCKO v2 Pooled library contains over 100,000 unique gRNAs targeting 19,052 human genes (Sanjana et al., 2014). The lentiviral plasmid library is originally purchased from Addgene and then amplified strictly following the manufacturer’s protocol (https://media.addgene.org/cms/filer_public/b5/fd/b5fde702-d02c-4873-806f-24ac28b2a15a/GeCKO v20_library_amplification_protocol_1.pdf).

***Note:*** GeCKO v2 library is delivered and amplified as two half-libraries (A and B). Combine the two plasmid half-libraries with a 1:1 ratio before making the lentiviral library.

2. Seed 293T cells into two 150 mm dishes at a seeding density of 5 × 10^6^ cells/plate with the antibiotic-free DMEM complete (no Penicillin/Streptomycin).
3. Grow the cells at 37°C with 5% CO_2_ and allow the cells to achieve 80% confluency.
4. Transfection of HEK293T cells using PolyJet transfection reagent is performed following the manufacturer’s protocol (https://signagen.com/DataSheet/SL100688.pdf) with some modifications.
  a. For each 150 mm plate, replace the medium with 20 mL of fresh antibiotic-free DMEM complete 30 min before transfection.
  b. For each 150 mm plate, prepare two clean 1.5-mL microcentrifuge tubes. Add 0.5 mL of DMEM (serum-free), 10 μg of psPAX2, 1 μg of pMD2.G, and 10 μg of GeCKO v2 plasmid library into one tube. Add 0.5 mL of DMEM (serum-free) and 48 μL of PolyJet into the other tube. Mix well by gentle pipetting respectively.
  c. Add the diluted PolyJet immediately into the diluted plasmids, pipette up and down 3–4 times, or vortex briefly to mix.
  d. Incubate the mix at 25°C for 10–15 min.
  e. Carefully transfer the mixture to the HEK293T cell culture dropwise and rock the dish to spread the mixture evenly. Put the cells back in the incubator.

5. Harvest GeCKO v2 lentiviral library.
  a. At 18 h post-transfection, replace the medium with 20 mL of fresh DMEM complete in each plate. Grow the cells for additional 30 h at 37°C in 5% CO_2_.
    ***Note:*** At this step, all operations should be as gentle as possible, as 293T cells may easily be detached from the plate.
  b. At 48 h post-transfection, collect the culture medium containing the lentiviral library to a 50 mL sterile centrifuge tube (low retention).
    ***Optional:*** To completely remove the detached cells and cell debris, centrifuge the tube at 500 g for 5 min. Then filter the supernatant through a 0.45 μm syringe filter to a new 50 mL centrifuge tube.
  c. Split the lentiviral solution into 1 mL aliquots in the 1.5 mL microcentrifuge tubes. Store the lentivirus in the −80°C freezer.

### Lentiviral titer estimation

**Timing: 5 days**

Below we provided a detailed step-by-step protocol for estimating the titer of the lentiviral library. A lentiviral library with high titer would be beneficial to generate a high-quality pooled cell library.

6. Seed HeLa-Cas9 cells into the 6-well plate with a seeding density of 0.3 × 10^6^ cells/plate and incubate the cells at 37°C with 5% CO_2_.
7. After 24 h, the cells should be ∼80% confluent. Thaw the GeCKO v2 lentiviral library on ice.
8. Prepare six 15 mL tubes and dilute the lentiviral library in DMEM complete with a 3-fold serial dilution (dilution factors of 3, 9, 27, 81, and 243, respectively). Leave one well without lentiviral infection as a control.
9. Remove the medium in the 6-well plate and transfer the diluted lentiviral library solution to each well.
10. After 12 h, replace the medium with fresh DMEM complete.
11. At 24 h post-transfection, change the medium with DMEM complete containing 2.5 μg/mL puromycin.
12. Incubate the cells with DMEM complete containing 2.5 μg/mL puromycin for 48 h. Trypsin digest and count live cell numbers with trypan blue staining.
13. Plot infected cell ratio (estimated by colony-forming method) versus dilution rate into a chart (Figure 3). The multiplicity of infection (MOI) is approximately equal to the infected cell ratio at low numbers (<20%).

**Figure 3:**
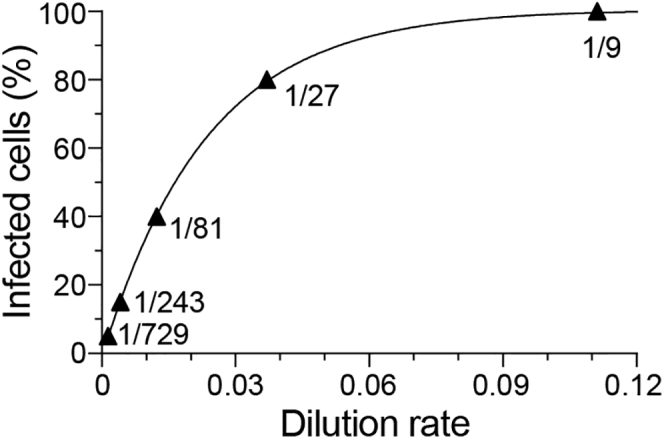
Estimation of MOI A representative plot of the percentage of infected cells versus dilution rate.
14. Estimate the lentivirus volume that can achieve an MOI of 0.3 for the next step (Shalem et al., 2014, 2015).

### Pooled cell library preparation

**Timing: 8 days**

Below we provided a detailed step-by-step protocol for the preparation of the pooled CRISPR cell library. We recommend checking the gRNA coverage of the starting cell library sample before starting the toxin screen, though it can also be performed by the end of the whole screen process.

15. Seed HeLa-Cas9 cells into four 15 cm dishes at a seeding density of 8 × 10^6^ cells/plate and incubate at 37°C in 5% CO_2_.
16. After 24 h of incubation, the cells would reach 80% confluent.
17. Thaw the GeCKO v2 lentiviral library on ice. Transfect the HeLa-Cas9 cells at an MOI of 0.3 and put the cells back in the incubator.

***Note:*** Add the lentiviruses dropwise into the cell culture, and rock gently three to four times to ensure the even separation of the viruses.

18. At 12 h post-infection, replace the medium with 20 mL of fresh DMEM complete.
19. At 24 h post-infection, replace the medium with 20 mL of DMEM complete containing 2.5 μg/mL puromycin to start the puromycin selection.
20. Keep the cells under 2.5 μg/mL puromycin for 48 h. Change the medium every 24 h.
21. Replace the medium with 20 mL of DMEM complete containing 1.25 μg/mL puromycin and incubate for additional 48 h. Change the medium every 24 h.

***Note:*** Passage cells into a new plate if the confluency is too high.

22. Trypsin-digest all cells and combined all cells. Freeze half of the cells at −80°C as a backup stock. For the rest of the cells, seed into three 15 cm dishes and allow to grow to 70% confluent.

***Note:*** The backup cell stock can be resuscitated as starting cell library.

23. Harvest one plate of cell libraries and save it as the starting library sample (R0). Proceed to the toxin screen with the other two plates.

### TcdB4 screen on HeLa-Cas9 cell library

**Timing: 5–6 weeks**

**Figure 4:**
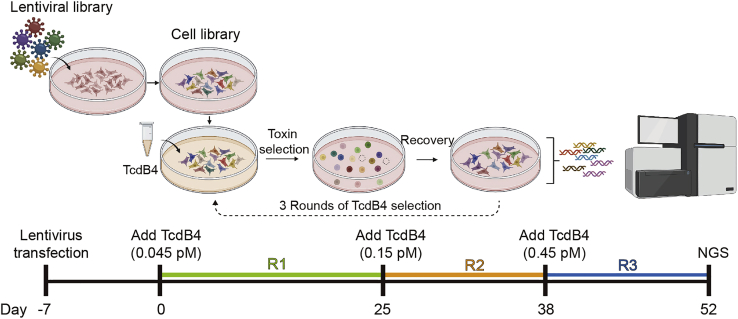
Timeline of the TcdB4 screening procedure Created with BioRender.com.

In this part, we selected the genome-wide CRISPR cell library with TcdB4 for three rounds. Each round takes about 2–3 weeks. A timeline of the screening procedure is shown in Figure 4.

24. Thaw TcdB4 stock on ice. Dilute TcdB4 in 40 mL of DMEM complete to a final concentration of 0.045 pM.
25. Replace the culture medium of the HeLa-Cas9 cell library with DMEM complete containing 0.045 pM TcdB4.
26. After 14 h, remove the supernatant and gently wash the cells 6 times with PBS to remove loosely attached round-shaped cells.
27. Trypsin-digest the rest cells and seed all cells back to the original plate. Add 20 mL of fresh DMEM complete to each dish and place the plates back in the incubator.

***Note:*** There’s no need to transfer the cells to a new dish.

28. After 6 h, wash the plate again with PBS to remove the unattached cells, Add 20 mL of fresh DMEM complete to each plate.
29. Use microscopy to check the condition of the cells until large cell colonies form (∼300 cells/colony). Change the medium with fresh DMEM complete if the spent turns yellow.

***Note:*** This step takes 2–3 weeks for the first round of screen and 1–2 weeks for the second/third round of screen.

30. Trypsin digest and combine all cells. Seed the cells into three new 10-cm dishes and allow them to grow to 70% confluent.
31. Harvest one plate of cell libraries and save it as the cell library sample for the first round of screen (R1). Proceed to the next round of screens with the other two plates.
32. Repeat steps 15–20 with 0.15 and 0.45 pM TcdB4 for the second and third rounds of the TcdB4 screen (R2 and R3). Collect the cell library samples with clear labels after each round of screen.

### PCR amplification of DNA sequences encoding small guide RNA (gRNA)

**Timing: 3 days**

Below we provided a detailed step-by-step protocol for obtaining the pooled gDNA fragments from the collected cell library samples.

33. Extract the genomic DNA from the cell libraries R0, R1, R2, and R3 using QIAGEN Blood & Cell Culture DNA Kit following the manufacturer’s instruction (https://www.qiagen.com/us/products/discovery-and-translational-research/dna-rna-purification/dna-purification/genomic-dna/blood-and-cell-culture-dna-kits).
34. A HeLa cell contains about 6 pg of genomic DNA. Each HeLa cell in the cell library is expected to harbor only one gRNA encoding gene. To ensure 20-fold coverage of the gRNA library (GeCKO v2 library contains over 100,000 gRNAs), 12 μg of genomic DNA is needed for the PCR amplification (6 pg of genomic DNA per cell X 100,000 gRNAs X 20-fold coverage).

***Note:*** R1, R2, and R3 are expected to contain much fewer numbers of gRNAs, a lower amount of genomic DNA may be used as the template for PCR recovering gRNAs encoding sequences from these samples.

35. For PCR amplification of DNA sequences encoding gRNAs, set up PCR reactions like the following:

**Table:** PCR reaction master mix

Reagent	Amount
DNA template	30 μg
Primer 1 (10 μM): lentiGP-1_F	10 μL
Primer 2 (10 μM): lentiGP-1_R	10 μL
2 × Taq (High-fidelity)	100 μL
ddH_2_O	Up to 200 μL

36. Run PCR reaction using the following conditions:

**Table:** PCR cycling conditions

Steps	Temperature	Time	Cycles
Initial denaturation	98°C	30 s	1
Denaturation	98°C	10 s	35
Annealing	50°C	20 s	
Extension	72°C	15 s	
Final extension	72°C	2 min	1

37. Agarose gel electrophoresis. Prepare a 2% agarose gel. Load 200 μL of PCR product for gRNA and run the gel.
38. Agarose gel extraction. Visualize the gel under Transilluminator (Figure 5). Cut the ∼200 bp band and purify DNA using an agarose gel extraction kit.

**Figure 5:**
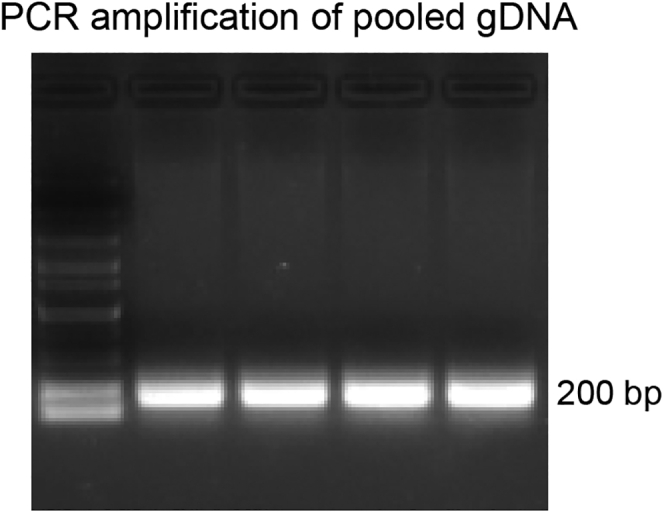
PCR amplicons of the pooled gDNA The PCR amplicons of the pooled gDNA are separated on a 2% agarose gel.
39. Quantify DNA and send for next-generation sequencing. 100 μg DNA yield is sufficient to perform the next-generation sequencing (NGS).

### NGS and data processing

**Timing: 1 week**

In this step, we perform NGS on Illumina Novaseq 6000 platform, align raw data to human GeCKO v2 library reference, use Cutadapt (Martin, 2011) to trim down raw sequencing reads and get the count number of each gRNA.

40. Library construction: 1 μg DNA per sample was used as input material for the preparation of libraries for NGS on the Illumina platform. Sequencing libraries were generated using NEB Next® UltraTM DNA Library Prep Kit for Illumina (NEB, USA) following the manufacturer’s recommendations (https://international.neb.com/protocols/2014/05/22/protocol-for-use-with-nebnext-ultra-dna-library-prep-kit-for-illumina-e7370). At this step, index codes were added to attribute sequences.
41. Sequencing: NGS is performed using an Illumina Novaseq 6000 platform at Novogene Bioinformatics Technology (China, https://en.novogene.com/). Sequencing cycles for read1/i7 index/i5 index/read2 are 150/8/8/150 respectively. 150 bp paired-end reads were generated. Around 2,000,000 reads for each sample (∼0.5 GB NGS data) were collected.

42. NGS data processing.
  a. Go to https://github.com/liuqinghe-007/Sequencing_results_analysis_of_CRISPR-cas9_screen.git.
  b. To align and count gRNAs in raw sequencing files, two perl scripts total_numbers_from_fq_file.pl and combine_a_b.pl, were written. Reference sequence files for human GeCKO v2 library (human_GeCKO v2_library_a.csv and human_GeCKO v2_library_b.csv) were downloaded from Addgene (https://www.addgene.org/pooled-library/zhang-human-gecko-v2).
  c. Forward sequencing primer: 5′-AATGGACTATCATATGCTTACCGTAACTTGAAAGTATTTCGATTTCTTGGCTTTATATATCTTGTGGAAAGGACGAAACACCG-3′. Reverse sequencing primer: 5′- GTTTTAGAGCTAGAAATAGCAAGTTAAAATAAGGCTAGTCCGTTATCAACTTGAAAAAGTGGCACCGAGTCGGTGCTTTTTTA-3′
  d. Raw sequencing reads are trimmed down to the 20-nucleotide gRNA sequence by Cutadapt version 4.0 (https://github.com/marcelm/cutadapt.git) and two files containing trimmed sequences are generated as trimmed_raw_reads.fq.gz.
  e. To obtain the count number for each gRNA, the script total_numbers_from_fq_file.pl is used to align trimmed sequences in trimmed_raw_reads.fq.gz to reference sequences in human_GeCKO v2_library_a.csv and human_GeCKO v2_library_b.csv files. This will produce two files including a_library_count.txt and b_library_count.txt. To launch the script, use the following bash command on the terminal:
    >perl total_numbers_from_fq_file.pl trimmed_raw_reads1.fq.gz trimmed_raw_reads2.fq.gz STEP1_OUTPUTDIR
  f. Use the script combine_a_b.pl to combine counts from A- and B- half-libraries via typing the command:

>perl combine_a_b.pl -i1 STEP1_OUTPUTDIR -o1 STEP2_OUTPUTDIR

43. The number of unique gRNAs and NGS reads for each gene are plotted in a chart.

**Figure 6:**
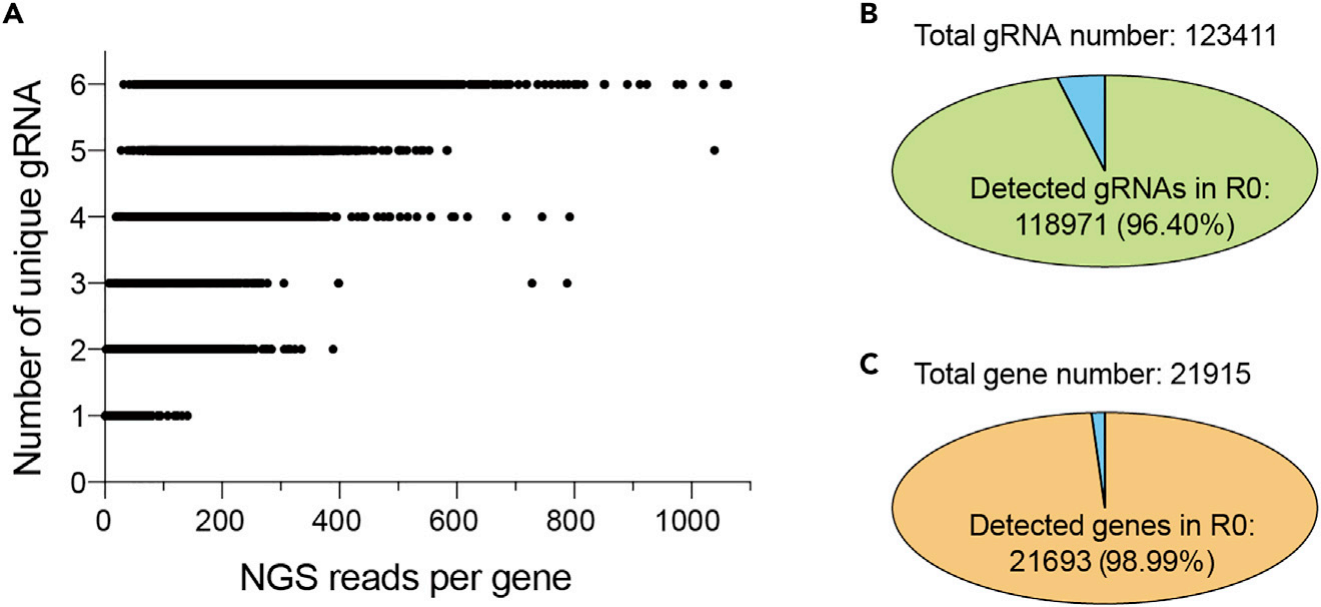
Quality control of the initial CRISPR knockout cell library (A) The number of unique gRNAs and the number of NGS reads for each gene in R0 are plotted in a chart. (B) The number of gRNAs/genes in R0 versus the number of total gRNAs/genes, as shown by breakdowns. (C) The number of identified genes in R0 versus the number of total targeted genes in the GeCKO v2 library.

***Note:*** Quality control of the initial CRISPR knockout cell library (R0) is very important. In a high-quality cell library, the gRNA/gene coverage should be as high as possible, while the number of NGS reads per gRNA need to be even. An example of gRNA/gene coverage in R0 is shown here (Figure 6).

## Expected outcomes

**Figure 7:**
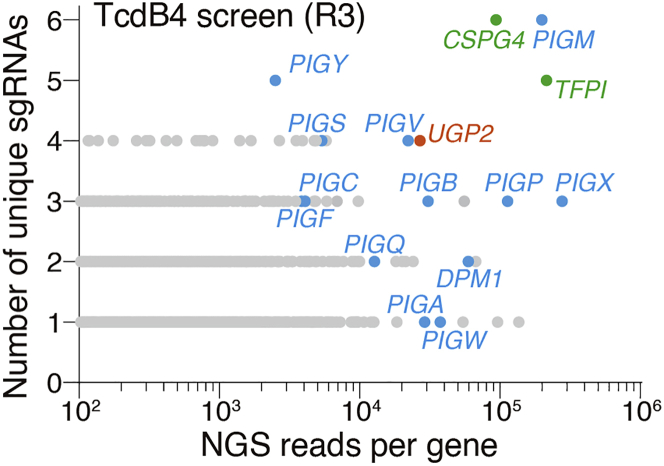
Genes identified from the TcdB4 screen (R3) as a screen outcome example The x-axis shows the number of NGS reads per gene, and the y-axis represents the number of identified gRNAs for each targeted gene.

Using this genome-wide CRISPR screen protocol in HeLa-Cas9 cells, we can uncover several host cell genes involving major steps in the TcdB4 toxin action, including cell surface recognition, endocytosis, acidification of the endosome, and production of the co-substrate UDP-glucose. An example of outcomes after three rounds of TcdB4 selection is shown in Figure 7. We have successfully unveiled two TcdB4 receptors, one enzyme responsible for producing UDP-glucose, and multiple receptor-related genes (Figure 7). For detailed outcomes, please refer to the original article (Luo et al., 2022).

## Limitations

This protocol provides detailed materials and reagents for the loss-of-function screen on HeLa cells and can be modified to other cell lines or cytotoxins. We chose HeLa cells in this protocol because HeLa cells are sensitive to TcdB4 in the cell rounding assay. But this protocol can be modified and applied to other cell lines. As a loss-of-function CRISPR screen, only functional but non-essential genes expressed in the model cells will be targeted. Also, gene redundancy could reduce the screening efficiency, therefore genes with multiple homologs may not be easy (but still possible) to be targeted.

## Troubleshooting

### Problem 1

Lentivirus integration efficiency on HeLa-Cas9 cells is much lower than the WT HeLa cells (before you begin step 8).

### Potential solution

When making the HeLa-Cas9 stable cell line, avoid infecting cells with high MOI.

### Problem 2

The screen results show a bias when using the resuscitated cells as starting cell library (step 22).

### Potential solution

Always perform the quality control for the starting library (R0). If the gDNA coverage is low in R0, regenerate the starting cell library.

### Problem 3

The numbers of cytopathic cells induced by the toxin are not consistent when the assays are performed in the 12-well plate and 15-cm dish (step 26).

### Potential solution

Usually, toxin concentration tested in a 12-well plate can be used directly in a 15-cm dish screen. However, if the cell rounding rate in a 15-cm dish appears different from the 12-well plate test, you can adjust toxin concentration. For instance, if very few cells survive in a given toxin concentration, try lowering the toxin concentration by 2-fold.

### Problem 4

Cells get contaminated by germs during the long-term screening (step 32).

### Potential solution

Sterilizing all reagents and materials before use. In particular, sterilizing the culture medium after adding the bacterial toxins by filtration through a 0.22 μm pore.

### Problem 5

NGS shows that the gRNA coverage in R0 is lower than expected (step 43).

### Potential solution

Don’t use viruses of low titer for R0 cell library preparation.

Optimized virus package conditions as the following:

• Use 293T cells with low passage numbers.
• Estimate transient transfection efficiency with a reporter plasmid before making the library. If plasmid transfection efficiency is low, transfect using high-quality DNA and adjust the transfection reagent-DNA ratio.

## Resource availability

### Lead contact

Further information and requests for resources and reagents should be directed to and will be fulfilled by the lead contact, Liang Tao (taoliang@westlake.edu.cn).

### Materials availability

All unique/stable reagents generated in this study are available from the lead contact with a Material Transfer Agreement.

## Data Availability

The code generated during this study is available at GitHub: https://github.com/liuqinghe-007/Sequencing_results_analysis_of_CRISPR-cas9_screen.git.
